# A hybrid tunable THz metadevice using a high birefringence liquid crystal

**DOI:** 10.1038/srep34536

**Published:** 2016-10-06

**Authors:** Nassim Chikhi, Mikhail Lisitskiy, Gianpaolo Papari, Volodymyr Tkachenko, Antonello Andreone

**Affiliations:** 1 University of Naples “Federico II”, Department of Physics “Ettore Pancini”, Naples, 80125, Italy; 2 CNR-SPIN, UOS of Naples, Naples, 80126, Italy; 3 CNR-ISASI, Pozzuoli (Na), 80078, Italy

## Abstract

We investigate a hybrid re-configurable three dimensional metamaterial based on liquid crystal as tuning element in order to build novel devices operating in the terahertz range. The proposed metadevice is an array of meta-atoms consisting of split ring resonators having suspended conducting cantilevers in the gap region. Adding a “third dimension” to a standard planar device plays a dual role: (i) enhance the tunability of the overall structure, exploiting the birefringence of the liquid crystal at its best, and (ii) improve the field confinement and therefore the ability of the metadevice to efficiently steer the THz signal. We describe the design, electromagnetic simulation, fabrication and experimental characterization of this new class of tunable metamaterials under an externally applied small voltage. By infiltrating tiny quantities of a nematic liquid crystal in the structure, we induce a frequency shift in the resonant response of the order of 7–8% in terms of bandwidth and about two orders of magnitude change in the signal absorption. We discuss how such a hybrid structure can be exploited for the development of a THz spatial light modulator.

The enormous progress in the field of metamaterials paved the way in recent years to a new era for novel devices able to realise exotic electromagnetic responses, some of them impossible to achieve with natural materials, ranging from negative refraction^1^ and superlensing^2^ to enhanced transmission^3^, perfect absorption^4^, cloaking^5^,^6^ and, more generally, to those properties based on coordinate transformation design^7^. The electric and magnetic responses achieved by metamaterials derive microscopically from the geometry of their unit cells and are preserved in the macroscopic medium. This is analogous to the overall properties of a conventional material, which are determined not only by the nature of the constituent atoms (its chemical composition) but also by a strong dependence on the lattice structure. Since their artificial structure is scalable to operate at nearly any frequency, metamaterials presently represent a bottom-up design paradigm for the construction of advanced electromagnetic materials spanning the frequency spectrum.

Initially, most of the metamaterial structures and devices were implemented at microwave frequencies because of the easiness in sample preparation and characterization^8^. Fabrication of sub-wavelength unit cells becomes increasingly challenging in moving from microwaves to higher frequencies, and only recently significant progress has been achieved towards the development of metamaterials operating in the THz region via standard photolithography and metal deposition technologies^9^.

Besides fabrication problems and tolerances, many applications of metamaterials are hampered by the lack of tuning capabilities and limited working bandwidth, which are mainly due to the inherently resonant nature of the sub-wavelength structures^10^. In the THz region specifically, the possibility to manipulate radiation in real-time can have enormous applications such as short-range wireless communication and ultrafast switches/modulators, besides helping in reducing the so-called “THz gap”. Tunable metamaterials refer to metamaterials with tunable responses to the incident electromagnetic waves. In these artificial structures, the interaction with the electromagnetic field can be manipulated, in order to get control of properties such as polarization, directionality, and amplitude. A tunable or reconfigurable metamaterial therefore stems its operation from the influence that an intentional change in, for example, the unit cell circuit, the constituent material properties, or the geometry, may have on its electromagnetic behaviour. In contrast to natural materials, where modification of their properties through tuning is somehow limited by the chemical bonding and by the intrinsic nature of the atoms inside the crystal lattice, the range of tunability for metamaterials is much broader since the stimulus provided by the “lattice” can be made much stronger by an appropriate design.

Tunable metamaterials can be classified into two wide categories, depending on the tuning mechanism. The first category includes those structures based on structural reconfiguration, that is on the geometrical changes induced by mechanical shifting or deformation of the metamaterial such as lattice or unit cells reshape, rotation or bending^11^. Even if structural tunability has a great potential to reduce many of the complications with passive metamaterials, this is however done at the expense of increasing fabrication complexity and cost. The other category is based on changing the effective electromagnetic properties via various linear and nonlinear effects in the sub-wavelength resonators^12^ or in the substrates^13^. Here the mechanism relies mostly on the transformation induced in the constituent materials or in the surrounding media by an external excitation like optical pumping^14^, magnetostatic field^15^, bias voltage^16^, electrical or thermal effects^17^. Many materials, such as liquid crystals (LC)^18^, phase change materials^19^, III–V semiconductors^20^ etc, have been shown to be effective in developing prototypal devices, and some of them are possible candidates for wide use of tunable metamaterials.

So far, the majority of the work on active metamaterials covering the THz spectrum has been done on single-layer planar composites patterned on insulating or semiconductor substrates such as silicon or gallium arsenide, for a designed resonant response of the electrical permittivity or magnetic permeability.

Very recently, however, some works have been carried out on the use of electrically controlled liquid crystals to realize frequency tunable metamaterial absorbers^21^, spatial light modulators^22^ or transducers^23^ in the THz region. It has been demonstrated that, using standard planar geometry and a high birefringence LC, a frequency shift up to 4% and an overall modulation depth ranging from 20% to 50% can be realised. In all these structures, the main difficulty for the realisation of devices with larger tunability lies in the planar metamaterial geometry, which impedes to use the LC birefringence at its best.

In this paper we present the design, simulation, fabrication and characterization of a tunable hybrid metadevice operating at around 1 THz, which can achieve a relatively large frequency shift using a nematic liquid crystal as the tuning mechanism. This translates in a signal modulation depth much stronger than previously published results, close to 100% within a reasonable bandwidth. The main novelty here consists in the design, which is based on a three-dimensional geometry capable to enhance the inherent response of the single unit cell and at the same time to improve the efficiency in modulating and/or steering the electromagnetic waves passing through the macroscopic medium.

## Results

### Metamaterial design

**Figure 1:**
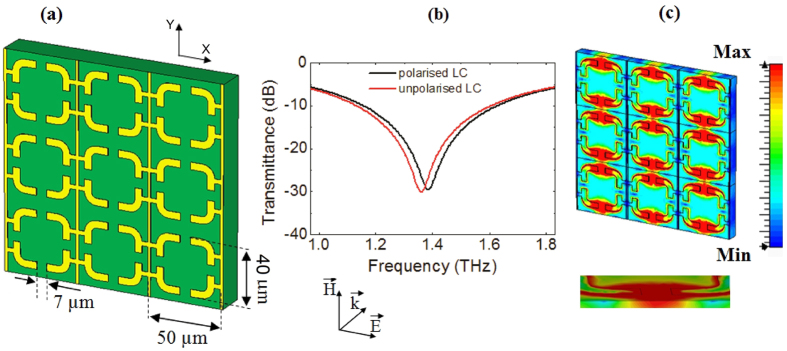
Two-dimensional metamaterial. (**a**) 3 × 3 array based on “four gap” SRR unit cells. Geometrical parameters are: unit cell size 50 μm, ring side 40 μm, ring width 5 μm, gap 7 μm. Metal thickness is 200 nm. (**b**) Simulated transmission response for the device with polarized (black curve) and unpolarized (red curve) LC. (**c**) Electric field distribution on the array surface (planar view) and in the gap region (transverse view) respectively. Intensity values are given in a pseudocolor scale.

The initial approach towards our hybrid device was the design and analysis of a simple tuning system operating at around 1 THz and composed of arrays of split-ring resonator (SRR) unit cells covered with liquid crystal. A SRR is basically a sub-wavelength magnetic resonator made of one or more concentric and inductive metallic rings loaded by capacitive gaps (Fig. 1(a)). Currents circulating in each ring give rise to a magnetic dipole moment, producing its own flux to enhance or oppose the incident field depending on the SRR resonant properties. As a consequence, an array of SRRs acts as an equivalent medium exhibiting a Lorentz-like permeability and a frequency band of negative μ values^24^.

It is important to note that under this configuration the current induction in the SRRs is caused by the electrical coupling between each unit cell of the array and the normally impinging THz wave. The “four-gap” design shown in Fig. 1(a) makes the structure more compact and responsive to the incident signal and at the same time symmetric along the two sides with regard to the polarisation of the electric field **E**. Another benefit is that it is easy to induce an arbitrary change in the frequency response by adding any kind of intrinsic or extrinsic stimulus to the overall capacitance of the metamaterial. This can be realised by inserting a nematic liquid crystal in the area surrounding the four capacitive gaps, as it will be better explained later in the text.

The electrodynamic properties of the planar structure were simulated using a commercial electromagnetic code. In order to apply a bias voltage and to properly polarize the LC inside the structure, thin conducting strips were added in the design of the planar SRR layer, connecting adjacent unit cells and running along the overall length of the meta-device.

Under THz radiation, this hybrid metamaterial shows a limited frequency shift of the electromagnetic response when the LC is polarised^25^, and at the same time a low field confinement around the gaps area, which translates in a relatively strong signal crosstalk between adjacent cells (see Fig. 1(b,c) respectively).

**Figure 2:**
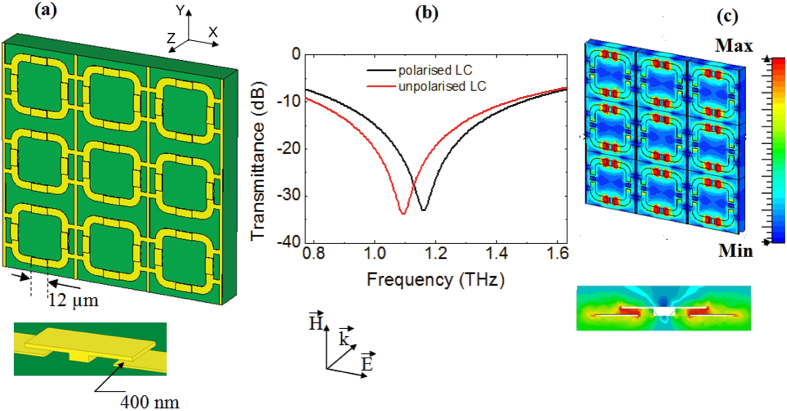
Three-dimensional metamaterial. (**a**) 3 × 3 array based on “four gap” SRR unit cells with stand up capacitors. Geometrical parameters in the plane are the same as in Fig. 1. At the centre of each gap, there is a pedestal with a rectangular 2.5 μm × 7 μm shape, and two cantilevers forming the suspended structure with 12 μm overall length and 600 nm thickness. The vertical gap distance is 400 nm. (**b**) Simulated transmission response for the device with polarized (black curve) and unpolarized (red curve) LC. (**c**) Electric field distribution on the array surface (planar view) and in the gap region (transverse view) respectively. Intensity values are given in a pseudocolor scale.

To optimize the performance of the SRR array with LC, one of the most effective ways is to modify the induced currents circulating through the capacitive gaps. For an efficient tuning, our idea has been to create controlled vertical capacitors over the ring gaps (since they cover the most sensitive area), and use the LC to change the permittivity of those capacitors. Therefore, suspended metallic caps have been designed in order to have cantilevers that overlap each side of the ring gaps (Fig. 2(a)), so that standing capacitor armatures can be formed and filled with LC as dielectric medium. As it will be shown in the next section, simulations indicate that these suspended structures are mostly responsible for the variation of the overall device capacitance, affecting the metamaterial electromagnetic response and producing a multi-fold increase in the frequency shift compared with the two dimensional analogue.

### Simulation

The working principle of our three-dimensional hybrid metadevice is based on the use of liquid crystal as tuning element. A proper modelling of the LC is the key element for the successful simulation using any electromagnetic software tool.

Simulations were performed using CST™, a commercial electromagnetic code. We chose as tuning element a liquid crystal having an ordinary refractive index n_o_ = 1.62, an extraordinary refractive index n_e_ = 1.83, and thus a birefringence of 0.21. These values refer to the properties (@ 1 THz) of the compound LCMS-107, which is a mixture of isothiocyanates having rod-shaped molecules and exhibiting a transition to the isotropic phase at T_c_ = 110 °C^26^.

LCs with an isothiocyanato terminal group are known to exhibit a nematic phase over a broad temperature range with the highest birefringence, up to 0.5 at the visible wavelength^27^, and rather small losses at GHz and THz frequencies^28^. This LC was purchased from LCMS Ltd Co. (Winter Springs, FL, USA) and has been actually used for the experimental characterization of the hybrid metadevice. Under alignment conditions, nematic liquid crystals have a preferential direction for their molecules, which defines the director axis. As a result of the uniaxial anisotropy, the electric component of the THz radiation experiences a different dielectric constant when oscillating in a direction parallel (extraordinary) or perpendicular (ordinary) to the director.

We assume that originally, at room temperature and in the tiny spatial region under consideration (the volume between the SRR base electrode and the suspended cantilevers), the LC director is parallel to the meta-device plane. This is in agreement with the alignment of a nematic LC confined within cylindrical pores of similar lateral dimensions in aluminium oxide^29^ (note that aluminium surfaces are naturally oxidized). In a liquid crystal with positive dielectric anisotropy Δε this kind of anchoring between the LC and the smoothed oxide surface is caused mainly by induced dipole-dipole interaction derived from Van der Waals force^30^. LC molecules must be preferably oriented along the metadevice SRR sides in order to keep the director undistorted in the regions close to the edges shared by vertical and horizontal walls of the electrodes. Applying an external bias voltage across the liquid crystal layer induces an electric field that reorients the molecules along the z-axis. Under the assumption that this voltage be much larger than the Fréedericksz transition threshold^31^, we can safely state that liquid crystal is fully polarised but for a very thin layer close to the aligning surfaces. This ensures an effective modulation of the resonance frequency.

To model the tuning properties of the three-dimensional metamaterial, we consider the LCMS-107 as an anisotropic material along the three axes, with the real part of its dielectric permittivity given in a vectorial form as 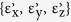.

In the area near the meta-device gaps, the THz electric field is strongly enhanced in the plane, in the direction normal to the gap themselves^32^. In the reference frame we chose, this direction coincides with the x-axis.

In our simulations, the highest possible tunability is achieved when the LC director is initially aligned along the x-axis, since in such a way the medium exhibits the largest dielectric anisotropy when a bias voltage is applied in the z-direction. The LC layer (real part) permittivity is therefore considered as {3.35, 2.62, 2.62} and {2.62, 2.62, 3.35} without and with an external electric field, respectively.

For the sake of clarity for the main argument, that is the study of the shift in the frequency response, the effect of losses in the SRR array and in the LC is not taken into account in the simulation of the metamaterial, which is therefore treated at a first stage as a perfect conductor in a homogeneous medium with zero absorption coefficient.

Following these considerations, we can simulate the transmission response of the three dimensional meta-device depicted in Fig. 2(a) and its two-dimensional analogue of Fig. 1(a), assuming that an incident electromagnetic wave impinges the surface with a propagation direction perpendicular to the SRR plane. Curves for the polarised and unpolarised LC are presented and compared in Figs 1(b) and 2(b) for the 3D and 2D device respectively. Firstly, one can observe that in both configurations there is a strong absorption dip, which is the result of the electric coupling to the SRR magnetic resonance produced by the THz wave^33^. This dip is more pronounced for the three dimensional geometry, showing that the presence of the cantilevers actually doesn’t affect the coupling but on the contrary enhances the resonant oscillation of the induced currents inside each SRR unit cell. Moreover, as expected, the resonance frequency for the SRR base layer only is higher than the value for the full hybrid structure because of the absence of the vertical capacitors (cantilevers), which reduces the overall effective capacitance of the metamaterialbased device. For both cases, results show that the LC polarization using an external voltage produces a frequency blue shift, induced by the permittivity decrease in the THz field direction. In the two-dimensional structure, however, the shift is less than 2%, whereas for the three dimensional geometry the response enhancement in the spatial region under consideration multiplies the effect, translating in a frequency shift up to 8% around the central frequency at 1.1 THz.

Besides the increase in the frequency tunability, another advantage of such structure is related to the crosstalk. In fact, a strong field confinement is observed in the stand up capacitors inserted on the SRR gaps, with an obvious reduction in the coupling between adjacent units cells and therefore a superior spatial selectivity (see Fig. 2(c)) with respect to the simple planar device shown in Fig. 1(a).

### Measurements

**Figure 3:**
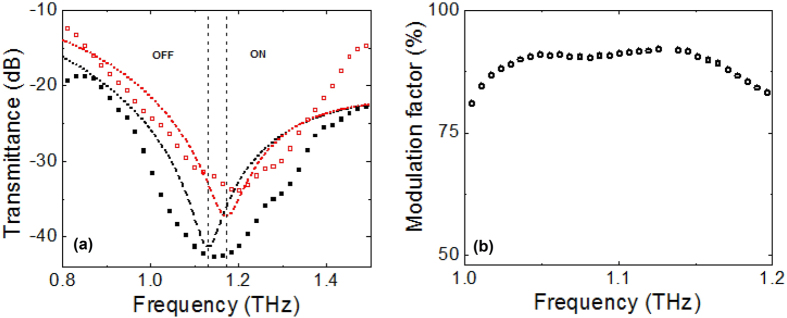
(**a**) Transmission spectra of the hybrid metamaterial measured at zero bias (state OFF, full black square points) and at 10 V (state ON, open red square points). Dashed curves refer to the results of simulations (OFF, black line; ON, red line), assuming a LC not perfectly aligned with the THz field in the unpolarized state and with a complex anisotropic dielectric permittivity (see text for details). Dashed vertical lines highlight the frequency blue shift switching the metadevice from OFF to ON. (**b**) The measured percentage modulation factor as a function of frequency in the interval 1.0–1.2 THz.

The fabricated metamaterial is based on an array of SRR unit cells with an effective area of 3 × 3 mm^2^. Figure 3(a) shows the comparison between the normalised transmission spectra of the hybrid structure measured at 0 V (state OFF) and when the 10 V bias voltage is applied (state ON), respectively. In both cases, a dip is observed in the range 0.9–1.4 THz, indicating that the insertion of the metadevice in the beam path produces a strong resonant absorption of the THz electromagnetic wave. One can also note that the experimental curves are somehow wider and more asymmetric in comparison with what is expected from simulations. This might be ascribed to the presence of inhomogeneity in the structure, mostly related to an uneven height of the vertical capacitors. The nominal thickness of each cap is 400 nm, but because of the complex photolithographic process it may be lower in a certain percentage of unit cells, giving as a result a broadening of the resonance, especially at the higher part of the spectrum.

The initial assumption that the LCMS-107 mixture is in the nematic phase at zero bias is confirmed by the experimental observation of a relatively large Fréedericksz transition threshold. Increasing the bias voltage along z, we measured a threshold value around 1.5 V, from which we can estimate a corresponding electric field of the order of 15 KV/m in the region beneath the cantilevers, in line with previously reported values for similar nematic LCs^34^.

As a result of the field induced LC polarisation, in the transmission response there is a clear and almost rigid shift of the resonance curve from ≈1.09 THz to ≈1.17 THz. The frequency change between the two minima is larger than 7%, very close to what expected from the model. Also, when the LC director is aligned along the z-axis, an increase in the signal transmission is observed, especially at the higher frequencies.

We believe that the discrepancy between the ideal model and the experimental results is based on different effects that have not been taken into account in the simulations presented in Fig. 2(b).

Firstly, the response of the real metadevice structure might be well influenced by mechanisms that cannot be predicted with ease.

In the absence of any external stimulus the LC molecules are lying close to the device plane because of the surface anchoring, but not necessarily all oriented along the THz electric field direction, as in our starting assumption. A possible picture is that at zero bias the capacitance of the three dimensional unit cell is primarily determined by an effective real permittivity value *ε*_*eff*_ in the x-z plane, according to the formula^35^:


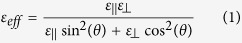


where θ is the angle between the THz electric field and the LC director, *ε*_‖_ and *ε*_⊥_ are the real part of the permittivity along and perpendicular to the director, respectively. Best results are obtained for θ ≈ 10°, yielding from Eq. (1) an effective value of the in-plane permittivity around 3.3.

An alternative explanation is the presence of a gradient layer where the LC director tilts and changes from its value at the surface to the one in the bulk volume under the applied tension. We estimate its thickness be about 10% of the LC gap distance^36^. As a matter of fact, the presence of such layer might well explain the slight disagreement observed between CST simulations and measurements.

A third effect one might consider is cantilever deformation, induced either by the capillary forces exerted by the infiltrated LC or by the low frequency driving field, since this would affect the spacing and as a consequence the capacitance seen by the THz wave, modifying the device response.

However, full 3D simulations (not reported here) show that frequency change in the device response due to a reduction in the spacing between the cantilever and the SSR is much higher than the effect produced by the LC polarisation, and it works in the opposite direction, that is it forces the device to operate at much lower frequencies than what is experimentally observed. Indeed, for the two LC states (OFF and ON) the simulation results are consistent with a gap of around 400 nm and possibly a slight deformation of few nm, causing the central frequencies to lower down just a little bit. Therefore, our understanding is that there is no meaningful frequency change caused by cantilevers displacement.

Lastly, the effect of electromagnetic wave absorption and its dependence on the LC director must be considered. In the microwave and millimetre wave range, losses in LCMS-107 are small and comparable, and the LC mixture can be treated as a homogeneous medium with negligible absorption. In the THz region, however, losses cannot be neglected, and besides that the ordinary component of absorption α_o_ grows faster that the extraordinary component α_e_, increasing by almost a factor 5 at around 1.5 THz^26^. This behaviour may partially explain the experimental observation of a damped resonance when the LC director is aligned along the z-axis, since in this case the THz electric field is mainly probing the strongly absorbent α_o_ term. In the same graph of Fig. 3(a), the results of simulations taking into account the presence of both planar anisotropy and losses in the liquid crystal are also presented. For the sake of simplicity, we assume that on average the α_o_ term is threefold larger than α_e_ = 20 cm^−1^ in the frequency range of investigation. The dashed black and red curves show the evaluated resonances of the meta-device assuming a complex anisotropic dielectric permittivity for the state OFF and ON respectively. Frequency minima are nicely reproduced, indicating that a model where LC molecules can be described with an imperfect alignment (θ ≈ 10°) with respect to the THz field works reasonably well. As for the loss contribution, the different absorption of the structure in the ordinary and extraordinary orientations manifests itself in a visible change of the resonance profile.

With a view to the application side, it is certainly useful to measure the ability of the hybrid meta-device to modulate the THz waves introducing the transmission modulation depth *M*, defined as


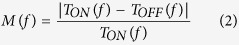


where *T*_*OFF*_ and *T*_*ON*_ represents the signal amplitude when no bias and 10 V tension is applied, respectively.

In Fig. 3(b) the parameter *M* as a function of frequency is plotted in the range 1.0–1.2 THz. In this interval, centred nearly the resonance frequency at zero bias, results show that the designed structure is capable to reach amplitude modulation values larger than 90% with a reasonably flat behaviour. Moreover, *M* values larger than 50% (3 dB bandwidth) are observed within a fairly large frequency band spanning from 0.9 to 1.4 THz.

## Discussion

We have designed, fabricated and experimentally demonstrated the capability of a hybrid metadevice having three-dimensional features and functionalised using a high birefringence LC to act as a spatial light modulator for THz waves. To achieve a larger frequency tuning, the standard metamaterial SRR geometry has been modified in such a way that the LC lies directly underneath the area, where the THz electromagnetic field is maximised and the externally applied ac electric field is the greatest. The tuning element is a nematic liquid crystal mixture, LCMS-107, belonging to the families with an isothiocyanato terminal group, that show the highest birefringence over a broad temperature range, the lowest viscosity amongst the liquid crystals with strong polarity and rather small losses at THz frequencies.

Simulations show that the artificial periodic structure we conceived, based on split-ring resonators with vertical capacitors, presents an extremely small electromagnetic coupling between adjacent meta-atoms, rendering this three dimensional metamaterial an ideal structure to realize a novel THz device with superior spatial selectivity.

THz transmission measurements are in excellent agreement with a model where the LC is described by introducing a complex dielectric permittivity tensor, with molecules in close proximity to the gap region characterised by a nematic order at zero bias. The reason for this assumption is the presence of boundaries in the lower and upper part of the Al structure, which favours the anchoring of the LC molecules to the metamaterial plane. Applying a small bias voltage at 1 KHz, we observe a significant blue shift of the response curve, close to 8%, and a relevant amplitude modulation, larger than 80% in a wide frequency bandwidth (0.2 THz) around the resonance frequency at 1.1 THz in the unpolarised case.

So far, no measured metadevices or metastructures functionalised using liquid crystal have shown similar performances. These results are comparable with modulation depth recently achieved on other systems: planar metamaterials electrically controlled using a Schottky gate structure (though at a fixed receiver angle and lower frequencies, ≈0.4 THz)^37^, or integrated systems consisting of a Quantum Cascade Laser and a graphene layer whose Fermi level can be dynamically tuned (operating however at higher frequencies, ≈3.2 THz)^38^.

The proper combination of a three-dimensional metamaterial and LC can be exploited to realise spatial light modulation in the truly THz frequency region, paving the way to the development of efficient pixelated arrays, where sub-modules of SRRs can be independently switched on and off using extremely small values of the bias tension. Moreover, the active component for the electrically controlled tuning is confined in the little volume included between each SRR gap and the corresponding cantilevers. Since thinner components enable to switch the device faster^39^, our geometry may potentially overcome the problem of slow modulation speed, which is usually one of the main obstacles in the development of LC-based meta-devices^40^.

## Methods

### Fabrication

**Figure 4:**
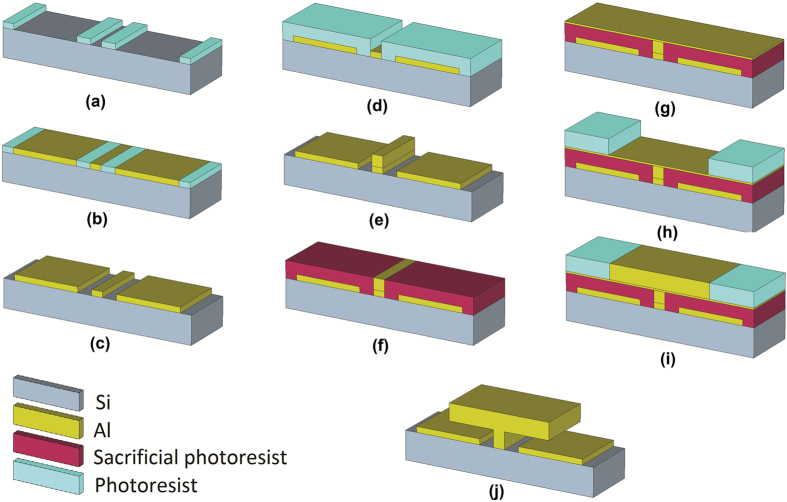
Schematic of the main fabrication steps: (**a**–**c**) base electrode; (**d**–**f**) pedestal; (**g**–**i**) cantilevers; (**j**) final vertical armature.

The metadevice is realized using a planar technology, based on DC sputtering deposition of an aluminium thin film on 1 × 1 cm^2^ n/PH doped Si substrate (381 μm thick) and standard UV photolithography. The challenge in making such type of structure obviously consists in the fabrication of the suspended cantilevers, located on each ring gap. We opted for a “mushroom” structure, as better specified below. The main steps in the device fabrication are schematically reported in Fig. 4. The process is divided into three main steps, namely to realize the SRR base electrode, the pedestal, and the cantilevers. As for the base electrode, an Al 200 nm thin film layer is patterned using a standard lift-off procedure (Fig. 4(a–c)). The realization of the suspended cantilevers (“mushroom”-like) consists of two parts. The first one is the fabrication of the pedestal used as a base to sustain the cantilevers. The procedure in this step is similar to the base electrode process but for the photomask used in the pedestal design (Fig. 4(d,e)). The fabrication of the cantilevers starts spinning a sacrificial photoresist and patterning it in order to guarantee electrical continuity to the upper conducting armature (Fig. 4(f)). Then, a very thin Al layer is deposited to protect the sacrificial photoresist (Fig. 4(g)) while performing a further photolithography for the geometrical definition of the suspended Al caps (Fig. 4(h)). After that, a 600 nm Al layer is deposited (Fig. 4(i)). The final step consists in the lift-off process and simultaneous removal of the sacrificial layer to get the 3D “suspended” structure (Fig. 4(j)).

The complete structure is an array of 3600 SRR-based meta-atoms with lattice parameter a = 50 μm. Each unit cell consists of a square ring with bended edges with 40 μm lateral sides, 5 μm width, and loaded with four capacitive gaps, two on each side, 7 μm wide. Running between adjacent unit cells are 2 μm wide conducting strips, connecting the array to a 1 × 1 mm^2^ single pad patterned in close proximity of the meta-device and operating as electrode.

**Figure 5:**
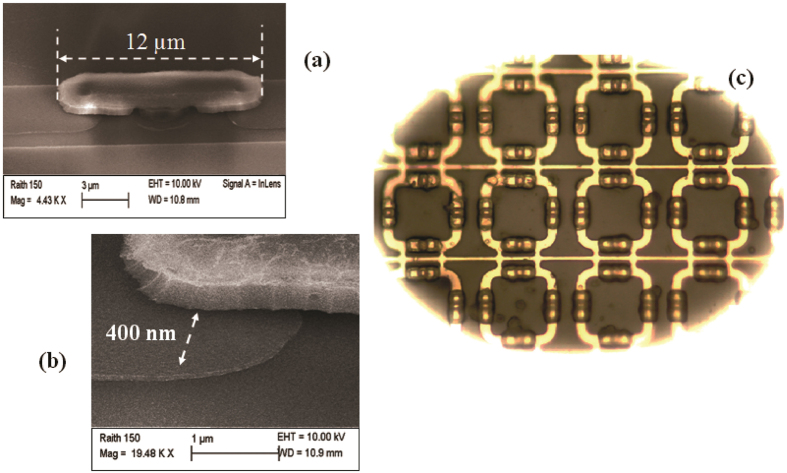
Details of the fabricated meta-device. SEM magnification of (**a**) the mushroom-shaped structure formed by two adjacent cantilevers on each side of the gap and (**b**) a single cantilever. (**c**) Optical image (×100) of the final hybrid meta-device after LC infiltration (and without the cover PET/ITO electrode).

At the centre of each gap, there is the vertical cap on a rectangular shaped (2.5 μm × 7 μm) pedestal, having 12 μm length and a distance of 400 nm from the upper surface of the planar structure. The scanning electron microscopy (SEM) images of a single standing vertical capacitor and of the gap between the base electrode and a single cantilever are reported in Fig. 5(a,b) respectively.

In order to effectively polarise the liquid crystal, a second electrode made of flexible polyethylene (PET) substrate (thickness of 127 μm) covered with a thin (75 nm) conductive Indium-Tin Oxide (ITO) layer (surface resistivity 100 Ω/sq) was put on the top of the structure. A mylar spacer 100 μm thick was used to create a distance between the Al cantilevers and the ITO electrode and protect the meta-device surface from crashing by mechanical contact. The presence of the dielectric spacer guarantees also the electrical insulation between the ITO electrode and the metasurface.

Under this configuration, the application of voltage between the continuously conducting ITO upper layer and the contact pad of the SRR array ensures a sufficiently uniform LC polarization in the region between the vertical caps and the planar gaps, where the THz electric field is maximized.

The nematic LCMS-107 mixture was inserted into the sandwiched structure using a needle tube to fill-up the three-dimensional metamaterial via capillary forces. To facilitate the infiltration process and to avoid contamination or voids inside the sub-unit cells, the process was undertaken under vacuum. Only a small LC volume is required to fill the entire near-field region of the metamaterial, which makes this three-dimensional structure ideal for tunability and easy integration in more complex circuits.

An optical microscope image (×100, top view) showing a detail of the final hybrid meta-device (without the cover PET/ITO electrode) is presented in Fig. 5(c).

The presence of liquid crystal in the area surrounding the vertical capacitors is clearly visible in the picture. The viscosity of the LC combined with the capillary forces causes the LC to be concentrated in the gaps areas and acts as physical support for the cantilevers too, preventing them from effective deformations under the influence of the applied ac field.

### Terahertz measurements

A THz-Time Domain Spectrometer (TDS) from Menlo Systems, based on a 1560 nm fiber laser with less than 90 fs pulse width and 100 MHz repetition rate, was used for the measurements.

In the standard setup, the laser output is split in 2 beams. Pump beam generates an electromagnetic transient (THz pulse, ≈1–2 ps) through the excitation of a low-temperature grown InGaAs/InAlAs–based photoconductive antenna (PCA) emitter, whereas the probe beam is used to detect the THz pulse using a similar PCA receiver. The variable delay between probe and pump beams, provided using a linear translation opto-mechanical stage, allows to detect the electric field amplitude of the THz pulse as a function of the timing difference.

Measurements were carried out on freestanding samples placed along the beam path with the THz electric field perpendicular to the metal connecting wires, in the focus of two Polymethylpentene (TPX) plano-convex lenses (effective focal length 54 mm). The spot diameter was about 2 mm, sufficient to uniformly illuminate the working area of the meta-device under test. Because of the strong tensile forces existing between the LC and the device surface at room temperature, the sample can be safely placed in the vertical position for the transmission measurements without any risk of leakage. The orientation of the LC molecules was electronically controlled by biasing the device with a 10 V peak-to-peak square waveform. We chose a modulation frequency of 1 KHz, which is optimal both for preventing the build-up of free carriers at the electrode metal interface and for the typical orientational relaxation time in nematic liquid crystals^41^.

**Figure 6:**
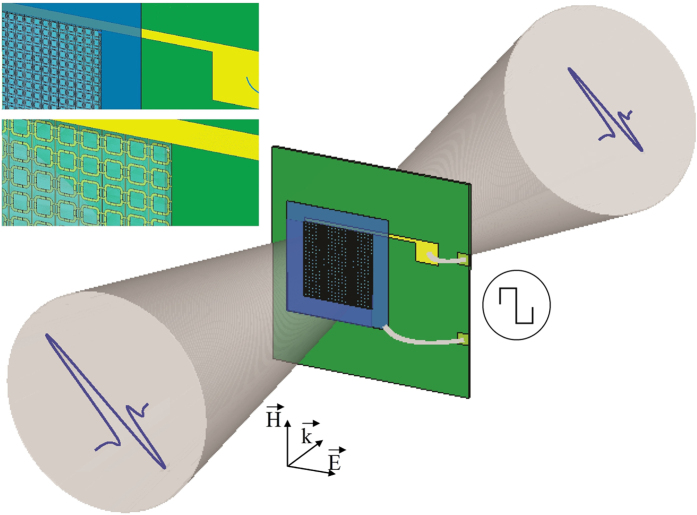
Sketch of the experimental set-up used to test the tunability of the metamaterial response.

A sketch of the experimental setup is shown in Fig. 6.

To accurately measure the metamaterial transmission spectrum, a bilayer consisting of a bare Si substrate and the ITO covered PET was used as a reference. The insertion of the structure in the beam path increases THz absorption, with a strong reduction of the Signal-to-Noise Ratio (SNR), making possible reliable measurements up to approximately 2 THz. Dividing the Fourier transform of the sample data by that of the reference data yields the transmission amplitude as a function of frequency. This simple procedure avoids complicate and laborious calibrations of the measurement setup.

All measurements were performed in a nitrogen-controlled dry atmosphere with nominally less than 0.1% humidity, to reduce or eliminate in the frequency spectra the water absorption lines caused by signal transmission in air.

The transmitted THz pulse presents echoes of the original wave because of the significant signal reflections induced by the presence of the LC/ITO interface^42^,^43^. Fabry-Perot resonances may complicate the analysis of the frequency spectrum, hiding or disturbing real spectral features with multiple ripples. In order to minimize the interference, a common strategy is to keep the scan length just short of the first echo or to apply a signal deconvolution to remove the echoes of the main pulse in the full time range, which works well but for the lowest amplitude signals. A combination of time windowing and numerical processing gives the best balance in results for the signal Fast Fourier Transform, in terms of both information content and frequency resolution.

## Additional Information

**How to cite this article**: Chikhi, N. *et al*. A hybrid tunable THz metadevice using a high birefringence liquid crystal. *Sci. Rep.* **6**, 34536; doi: 10.1038/srep34536 (2016).
